# Lack of FAM20A, Ectopic Gingival Mineralization and Chondro/Osteogenic Modifications in Enamel Renal Syndrome

**DOI:** 10.3389/fcell.2020.605084

**Published:** 2020-12-08

**Authors:** Victor Simancas Escorcia, Abdoulaziz Diarra, Adrien Naveau, Arnaud Dessombz, Rufino Felizardo, Vidjeacoumary Cannaya, Christos Chatziantoniou, Mickaël Quentric, Miikka Vikkula, Olivier Cases, Ariane Berdal, Muriel De La Dure-Molla, Renata Kozyraki

**Affiliations:** ^1^Centre de Recherche des Cordeliers, Sorbonne Université, INSERM, Université de Paris, Laboratory of Oral Molecular Pathophysiology, Paris, France; ^2^CRMR O-RARES, Hôpital Rothshild, UFR d’Odontologie-Garancière, Université de Paris, Paris, France; ^3^UMRS1155, INSERM, Sorbonne Université, Paris, France; ^4^Department of Human Genetics, De Duve Institute, Université catholique de Louvain, Brussels, Belgium; ^5^Institut des maladies génétiques, Imagine, Paris, France

**Keywords:** Fam20A, enamel renal syndrome, calcification, gingiva, fibroblast, osteoblast, cell death

## Abstract

Enamel renal syndrome (ERS) is a rare recessive disorder caused by loss-of-function mutations in *FAM20A* (family with sequence similarity 20 member A, OMIM #611062). Enamel renal syndrome is characterized by amelogenesis imperfecta, delayed or failed tooth eruption, intrapulpal calcifications, gingival overgrowth and nephrocalcinosis. Although gingival overgrowth has consistently been associated with heterotopic calcifications the pathogenesis, structure and interactions of the mineral deposits with the surrounding connective tissue are largely unknown. We here report a novel *FAM20A* mutation in exon 1 (c.358C > T) introducing a premature stop codon (p.Gln120*) and resulting in a complete loss of FAM20A. In addition to the typical oral findings and nephrocalcinosis, ectopic calcified nodules were also seen in the cervical and thoracic vertebrae regions. Histopathologic analysis of the gingiva showed an enlarged papillary layer associated with aberrant angiogenesis and a lamina propria displaying significant changes in its extracellular matrix composition, including disruption of the collagen I fiber network. Ectopic calcifications were found throughout the connective gingival tissue. Immunomorphological and ultrastructural analyses indicated that the calcification process was associated with epithelial degeneration and transformation of the gingival fibroblasts to chondro/osteoblastic-like cells. Mutant gingival fibroblasts cultures were prone to calcify and abnormally expressed osteoblastic markers such as *RUNX2* or *PERIOSTIN*. Our findings expand the previously reported phenotypes and highlight some aspects of ERS pathogenesis.

## Introduction

Enamel renal syndrome (ERS, OMIM #204690) or amelogenesis imperfecta type IG (OMIM #614253) is an autosomal recessive disorder characterized by enamel hypoplasia, delayed or failed tooth eruption, intrapulpal calcifications, and gingival overgrowth. Pulmonary calcifications, hearing loss, or vitamin D-deficiency associated hyperparathyroidism and amenorrhea may also be observed (Kantaputra et al., 2014; Pêgo et al., 2017). Nephrocalcinosis is systematically found in ERS patients; it is generally asymptomatic and associated with normal blood chemistry analyses. However, progressive renal dysfunction and failure have previously been reported (MacGibbon, 1972; Lubinsky et al., 1985; de la Dure-Molla et al., 2014).

We and others previously showed that bi-allelic loss of function *FAM20A* mutations cause ERS (Martelli-Júnior et al., 2008; Jaureguiberry et al., 2012; Wang et al., 2013; de la Dure-Molla et al., 2014; Kantaputra et al., 2014). *FAM20A* is a member of a small gene family of kinase-encoding genes that also includes *FAM20B* and *FAM20C*. FAM20B is a xylosyl kinase regulating proteoglycan synthesis whereas FAM20C is a Golgi casein kinase that phosphorylates most of the secreted proteins, including the enamel matrix ones, within Ser-X-Glu/pSer motifs (Hao et al., 2007; Tagliabracci et al., 2012; Zhang et al., 2018). FAM20A is thought to be a pseudokinase. FAM20A forms a complex with and allosterically activates FAM20C (Zhang et al., 2018) promoting phosphorylation of FAM20C targets. *FAM20A* displays a restricted expression pattern with high expression in larynx, testes and kidney (Nalbant et al., 2005). A strong FAM20A expression is also reported in the secretory and maturation stage ameloblasts, odontoblasts, dental pulp and gingiva of the mouse (O’Sullivan et al., 2011). Unlike FAM20A, FAM20C is ubiquitously expressed. Mutations in *FAM20C* cause the frequently lethal Raine syndrome (RNS, #OMIM259775) characterized by bone dysplasia and cerebral calcifications (Whyte et al., 2017). The oral findings of the non-lethal RNS forms are reminiscent of ERS and include gingival overgrowth and enamel hypoplasia (Acevedo et al., 2015).

Gingival overgrowth of variable severity is consistently found in ERS patients. Previously published histological data associate this finding with an increased amount of disorganized collagen fiber bundles, myofibroblasts, remnants of odontogenic epithelium, and psammomatous calcifications (Martelli-Júnior et al., 2008; Wang et al., 2019). However, the pathogenesis of the gingival calcifications, the formation and structure of the ectopic mineral deposits or their interactions with the connective tissue are largely unknown. We now report a novel case of ERS carrying a hitherto unreported null *FAM20A* mutation. In addition to the typical oral findings and nephrocalcinosis, ectopic calcified nodules are seen in the cervical and thoracic vertebrae regions. Immunomorphological and ultrastructural analyses of the affected gingival tissue indicate that the calcification process is associated with cells displaying a chondrogenic/osteoblastic-like phenotype. *In vitro* culture of the proband’s gingival fibroblasts confirms the histological data and suggests that the transdifferentiated cells enact cellular programs that mediate aberrant calcification. Our findings expand the previously reported phenotypes and highlight some aspects of the ERS pathogenesis.

## Materials and Methods

### Ethics – Patients Recruitment

An 18-year-old male patient was referred in 2014 for oral rehabilitation at the Reference Center of rare dental diseases (Rothschild Hospital, CRMR O-RARE, Paris, France). Diagnosis of ERS was based on clinical and radiological features (see section “Results”). This patient and healthy age-matched controls (*n* = 3) were recruited following informed consent in accordance with the principles outlined in the declaration of Helsinki. Written informed consent was obtained from the proband for the publication of any potentially identifiable images or data included in this article. The samples used were considered as operating waste according to the French law. Samples from the proband and controls were harvested during oral rehabilitation and were prepared for histological or cell culture analyses (authorization CODECOH DC-2018-3382).

### DNA Sequencing

DNA was purified from blood samples using a Dneasy Blood & Tissue kit (Qiagen, Germany). Exon-specific primers for all exons of FAM20A were designed as described in Jaureguiberry et al. (2012). PCR products bidirectionally sequenced with a Big-Dye Terminator v3.1 Cycle Sequencing kit (Applied Biosystems, CA, United States) and an ABI Prism 3130 Genetic Analyzer (Applied Biosystems).

### Immunocytochemistry and Histology

#### Immunohistochemistry

Approximately, 1 cm^3^ gingival samples from patients were fixed for 24 h at 4°C in 4% paraformaldehyde and then embedded in paraffin wax. After sectioning, epitope retrieval was achieved by heat. Sections were incubated for 2 h at room temperature or overnight at 4°C with primary antibodies; rabbit anti-Aggrecan (1/200; sc-28532; Santa-Cruz), rabbit anti-Calnexin (1/1000; EPR3632; ab92573; Abcam), rabbit anti-Cytokeratin 14 (1/750; ab15461; Abcam), rabbit anti-Calbindin D-28K (1/1,000; CB38, Swant), rabbit anti-FAM20A (1/250; OACD03385; Aviva), rabbit anti-Fam20C (1/250; OAAB01003; Aviva), rabbit anti-Periostin (1/500; ab14041; Abcam), rabbit anti-S100A9 (1/500; EPR3555; ab227570; Abcam), mouse anti-Alkaline Phosphatase (1/100; B4-78; R&D), mouse anti-alpha Smooth Muscle Actin (1/1000; 1A4; ab7817; Abcam), mouse anti-Keratan Sulfate (1/200; 4B3/D10; sc73518; Santa-Cruz Biotechnology), goat anti-FAM20A (1/200; sc-164308; Santa-Cruz Biotechnology), goat anti-VEGF (1/250; sc-1836; Santa Cruz Biotechnology), and rat anti-Procollagen type I (1/500; M-58; ab64409; Abcam). Secondary antibodies used were Alexa 488- or Cy3-conjugated donkey anti-rabbit (Jackson Immunoresearch Laboratories, West Grove, PA, United States; 1:500), Alexa 488- or Cy3-conjugated donkey anti-mouse (Thermo Fisher Scientific; 1:500), and Alexa 488- or Cy3-conjugated donkey anti-goat (Thermo Fisher Scientific; 1:500). Nuclear staining was achieved by 20 min incubation at room temperature in Hoechst 33342 (Thermo Fisher Scientific). Isotype controls were used as negative controls to help differentiate non-specific background signal from specific antibody signal (1/200; rabbit IgG, 02-6102; goat IgG, 02-6202; rat IgG1, 14-4301-82; mouse IgG1, 14-4714-82; mouse IgG2a, 14-4724-82, and mouse IgG2b, 14-4732-82, Thermo Fisher Scientific). Secondary antibodies were used as previously described. No cellular autofluorescence and no nonspecific labeling were detected in these conditions. Images were collected by confocal microscopy (Zeiss LSM8) and processed using ZEN (Zeiss) and ImageJ softwares.

#### Histology

Sections were stained with routine protocols for Haematoxylin and Eosin (HE), Picro-Sirius Red, Alcian Blue pH 1 and 2.5, Alizarin Red, von Kossa, and Goldner’s Trichrome stains.

### Western Blot

For western blot analysis (30 μg/sample) gingival extracts were used. Extracts were lysed in a PBS buffer (10 mMNaH_2_PO_4_, 150 mM Nacl, 6 mM Cacl_2_) with 1% Triton X-100 (Merck), 1 mM sodium orthovanadate, and Complete mini EDTA-free protease inhibitor cocktail tablets (Roche Diagnostics), pH 7.4. Immunoblotting analyses were performed by standard procedures using ECL reagents as described by the manufacturer (GE Healthcare). To standardize the protein expression across samples, we used an anti-GAPDH goat antibody at a dilution of 1/5,000. Western blot experiments were run in triplicate. Primary antibody was rabbit anti-FAM20A (1/250; OACD03385; Aviva).

### Fourier Transform InfraRed Microscopic Imaging

Three-μm-thick sections were collected on low emission slides (mirrir low-E, Kevley Technologies, IN). After de-paraffinization, Fourier transform infrared (FTIR) images were recorded with the commercially available SpotLight 400i FT-IR microscopy system from Perkin Elmer.

### Fibroblast Cell Culture

Control and proband gingival fibroblasts were established by plating small pieces of excised gingival on plastic dishes. Cells, particularly gingival fibroblast, migrate out of the explant and colonize the petri dish. The flasks were filled with Dulbecco’s modified Eagle’s medium-low glucose (DMEM) containing 20% fetal calf serum (FCS), 1% non-essential amino acid, penicillin/streptomycin (100 mg/mL) and amphotericin B (2 ng/mL). The flasks are then placed in an incubator programmed at 37°C in a humidity atmosphere at 5% CO_2_ and the cell culture medium was changed twice a week until the confluence of the cells (90% after about three weeks). Once at confluence, the gingival fibroblasts were trypsinized (Trypsin-EDTA, GIBCO^®^, 1 mL at 0.05%) and single-cell suspensions were seeded in 25 cm^2^ flasks containing DMEM 10% of FCS, passaged by splitting when they reached confluence, and frozen in liquid nitrogen until use. All primary cultured cells were used at passage 2. Mineralization assay: Mineralization was induced after 24 hours by addition of DMEM, 10% FCS, 1% non-essential amino acid, 1% penicillin/streptomycim, 0.5% amphotericin B, and 20 mM β-glycerol phosphate (Sigma). All cells in culture were subjected to 7 or 21 days of incubation in order to observe calcifications. Alizarin Red staining (ARS) was used to evaluate the presence or absence of calcifications. Fibroblasts monolayers in six-well plates were washed twice with PBS and fixed in 10% formaldehyde (Sigma) for 15 min at room temperature. Prior to the addition of 1 mL of 40 mM ARS (pH 4.1), distilled water was used to wash twice. Subsequently, plates were incubated for 20 min at room temperature with gentle shaking. Wells were washed four times with distilled water to remove excess of dye. Stained monolayers were visualized under phase contrast microscopy (Axiovert 135, Zeiss). Calcium forms an alizarin red S-complex, which is birefringent and is detected as orange–red precipitations by phase contrast microscopy.

### Transmission Electron Microscopy (TEM)

Gingival fibroblast cultures from patients were fixed for 48 h at 4°C in 2.5% glutaraldehyde solution in 0.1 M sodium cacodylate buffer. After washing with 0.1 M sodium cacodylate, samples were transferred to 2% osmium tetroxide for 2 h at 4°C. They are then dehydrated in ethanol at increasing concentrations and in propylene oxide. Then, we carried out an impregnation and the inclusion in resin Epon (Polysciences, Inc.). The resin is then polymerized at 37°C and then at 60°C. Sections were made using a Leica ultra-microtome (UCT). Semi-thin sections were stained with 1% toluidine blue and observed under an optical microscope. The zone of interest was carefully selected and seventy nanometers ultrathin sections were cut and deposited on gilder copper. The grids were stained with 1% uranyl acetate and lead citrate, and observed by TEM (Tecnai 12 electron microscope – CCD detector: SiS 1Kx1K Keen View).

## Quantitative RT-PCR

Gingival fibroblasts (10^6^) were seeded on six-well plates. When they reached early confluency, cells were cultured for three days in DMEM containing 10% FBS. For mineralization induction, the medium was changed to induction and considered day 1.

After 7 or 21 days of induction, total RNA was isolated using commercially available kits according to manufacturer guidelines (RNeasy Mini, Qiagen) and measured (Nanodrop, Peqleb). One microgram was used in a reverse transcription reaction (SuperScript First strand synthesis, Thermo Fisher Scientific). Quantitative-PCR was performed using Quantifast SYBR Green PCR Kit (Qiagen), reactions were performed in triplicate. Transcript levels were calculated using the standard curves generated using serial dilutions of cDNA obtained by reverse transcription of control RNA samples then normalized to HPRT. Primer sequences are for *RUNX2*, 5′-CCGGAATGCCTCTGCTGTTA and 5′-TGTCTGTGCCTTC TGGGTTC, *ALKALINE PHOSPHATASE*, 5′-CGTGGCTAAGA ATGTCATCATGTT and 5′-TGGAGCTGACCCTTGAGGAT, *PERIOSTIN*, 5′-TGCCCAGCAGTTTTGCCCAT and 5′-CGTT GCTCTCCAAACCTCTA, *COLLAGEN type 1 alpha1-chain*, 5′-AGGGCCAAGACATC-3′ and 5′-AGATCACGTCATCGCAC AACA-3′, and *COLLAGEN type 1 alpha2-chain*, 5′-CTGGT AGTCGTGGTGCAAGT-3′ and 5′-AATGTTGCCAGGCTCT CCTC-3′. The graphs plot the mean ± SD of the fold expression of three independent experiments performed each with triplicate samples. *T*-test statistical analysis showed significant differences at *p* < 0.01 (***), *p* < 0.05 (**), and *p* < 0.1 (*). Amplification specificities were assessed by melting curve analyses and amplicons were sequenced.

## Results

### Clinical and Genetic Diagnosis of Ers

**FIGURE 1:**
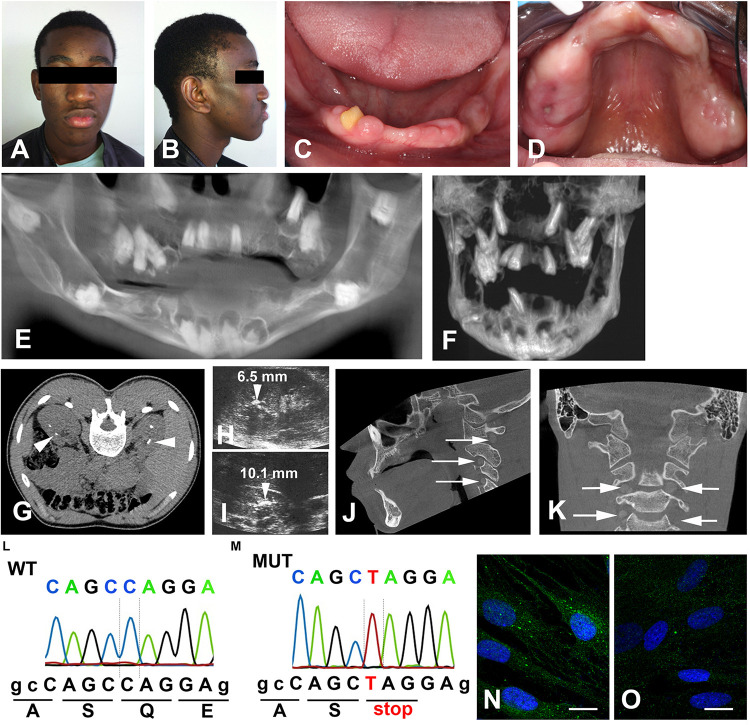
Clinical features of the proband. Front **(A)** and side **(B)** photos showing facial asymmetry, ocular hypertelorism and dense, bushy eyebrows. **(C,D)** Generalized gingival enlargement and a yellow–brown discoloration of the unique erupted tooth. **(E)** CBCT reconstruction; agenesis of 11, 14, 18, 28, 31, 36, 41, 42, 45, and 46; multiple unerupted teeth; large radiolucent pericoronal areas associated with impacted molars. **(F)** Mandibular and maxillary CBCT reconstruction. Note the displacement of the premolars into the maxillary sinus and the molars in the mandibular canal. **(G)** Renal axial CT-scan and **(H,I)** ultrasound showing bilateral medullary nephrocalcinosis (arrowheads). **(J)** Sagittal and **(K)** para-sagittal CBCT scans reveal round bilateral calcifications within the foramens of the cranial vertebrae (arrows). **(L,M)** Sequencing chromatograms of *FAM20A* exon 1 of a control **(L)** and proband **(M)**. The homozygous nonsense mutation, c.358C > T causes a premature termination at p.Gln120*. **(N)** Vesicular distribution of FAM20A in control gingival fibroblasts. **(O)** Loss of FAM20A staining in the proband’s gingival fibroblasts. Scale bars: **(O)**, *P* = 20 μm.

A 25-year-old male patient was referred to the Reference Center of rare oral diseases (Rothschild Hospital) as previously described (de la Dure-Molla et al., 2014). He was born to unrelated parents and family history did not reveal any ERS antecedents. Upon extra-oral examination facial asymmetry and bushy eyebrows were noted (Figures 1A,B). Intraoral examination showed presence of a yellownish-brown permanent mandibular right lateral incisor and generalized gingival enlargement (Figures 1C,D). The panoramic radiograph revealed agenesis of 10 teeth, failed eruption of the remaining ones as well as intrapulpal calcifications (Supplementary Figure 1A). Cone beam computed tomography (CBCT) showed aberrant locations of unerupted teeth, including within the rising branch of the mandible or invading the cortical mandibular bone (Figures 1E,F). Enlarged pericoronal spaces delimited by sclerotic margins, most likely hyperplastic follicles, were observed around several unerupted teeth (Figures 1E,F). Numerous calcified nodules were evidenced by renal CT scans, mainly localized around the cortico-medullary junction of both kidneys (Figure 1G). The presence of bi-lateral medullary nephrocalcinosis was further confirmed by renal ultra-sound (Figures 1H,I). Cone beam computed tomography and CT scans of the cervical and thoracic regions revealed curved, radio-opaque images consistent with calcium depositions at the level of the vertebral foramens (Figures 1J,K and Supplementary Figures 1B–D).

Blood chemistry analysis showed that the calcium level was at the upper limit (102 < 104 mg/mL) and that phosphate and creatinine levels were slightly increased (48 > 46 mg/L and 12.1 > 12 mg/L). Quantification of serum parathyroid hormone (PTH) was in a normal range (6.5 < 16.1 < 38.8 pg/mL). The clinical findings were reminiscent of ERS and this was confirmed by the identification of a novel, homozygous c.358C > T mutation in exon 1 (Figures 1L,M). This mutation introduced a premature stop codon (p.Gln120*), most likely resulting in a rapidly degraded truncated protein. FAM20A was indeed undetectable in gingival fibroblasts of the proband (Figures 1N,O). Gene analysis of his parents was not available.

### Fam20A and FAM20C Expression in the Control and Mutant Gingiva

**FIGURE 2:**
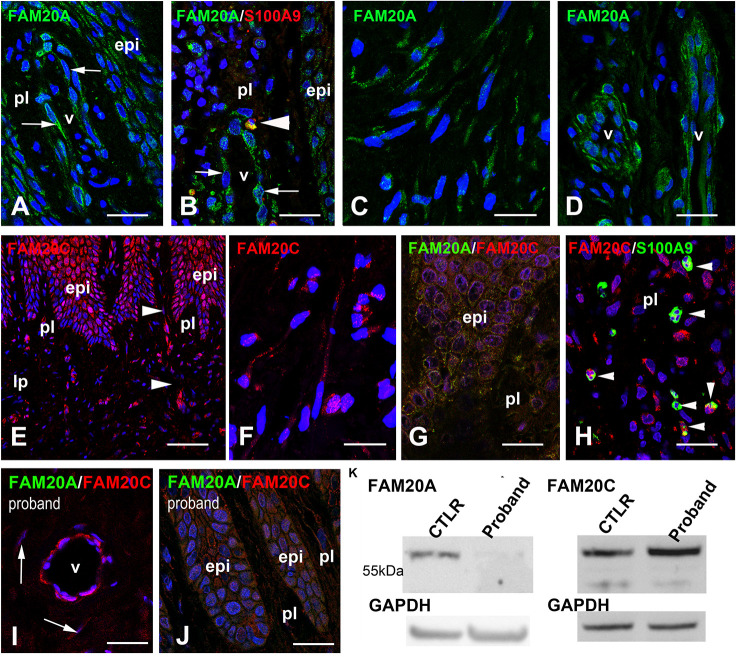
FAM20A and FAM20C expression in normal gingiva. **(A)** FAM20A expression is detected in epithelial cells (epi) and vascular cells in the papillary layer (pl). **(B)** FAM20A (green) is expressed in S100A9 (red) cells (arrowhead). Thin arrows bordered a blood vessel. **(C)** In fibroblasts, FAM20A is found in vesicles within elongated processes. **(D)** In the lamina propria, FAM20A is also expressed in endothelial and perivascular cells of blood vessels. **(E)** FAM20C distribution in epithelial, vascular (arrowheads) and fibroblastic cells of the papillary layer and of the lamina propria (lp). **(F)** FAM20C is found in vesicles within elongated processes of fibroblasts. **(G)** FAM20A (green) and FAM20C (red) are co-expressed in epithelial cells. **(H)** FAM20C expression in S100A9 positive populations (arrowheads). **(I,J)** Absence of FAM20A expression (green) in the proband’s gingiva; FAM20C (red) is detected in enlarged blood vessels **(I)** and gingival epithelium **(J)**. Arrows indicated FAM20C expression in fibroblasts. **(K)** Western-blot of FAM20A and FAM20C in control and proband gingiva. V: vessel. Scale bars: (**A**–**D,G**–**J)** = 100 μm; **(E)** = 200 μm; **(F)** = 20 μm.

The expression of FAM20A in the keratinocytes and endothelial cells of the human gingiva was previously reported (Wang et al., 2019). We confirmed the dotted/vesicular pattern of FAM20A in the epithelial and vascular cells of the control gingiva (Figures 2A,B). We additionally identified FAM20A co-expression with S100A9 in few cells (Figure 2B), a marker of macrophages and neutrophils (Wang et al., 2018). In the reticular layer of the lamina propria, the FAM20A signal was also seen in fibroblasts (Figure 2C) and vascular cells (Figure 2D).

The FAM20A paralog FAM20C was also detected in the gingival epithelium, vascular cells (Figure 2E) and fibroblasts (Figure 2F). FAM20C was co-expressed with FAM20A (Figure 2G) in epithelial cells. Like FAM20A, FAM20C was also expressed in few S100A9 positive cells (Figure 2H). In the proband’s gingiva, no FAM20A immunoreactivity was seen (Figures 2I,J). Despite the lack of FAM20A the distribution of FAM20C was globally the same and its electrophoretic profile was unmodified (Figures 2I–K).

### Histopathological Analysis of Mutant Gingiva

**FIGURE 3:**
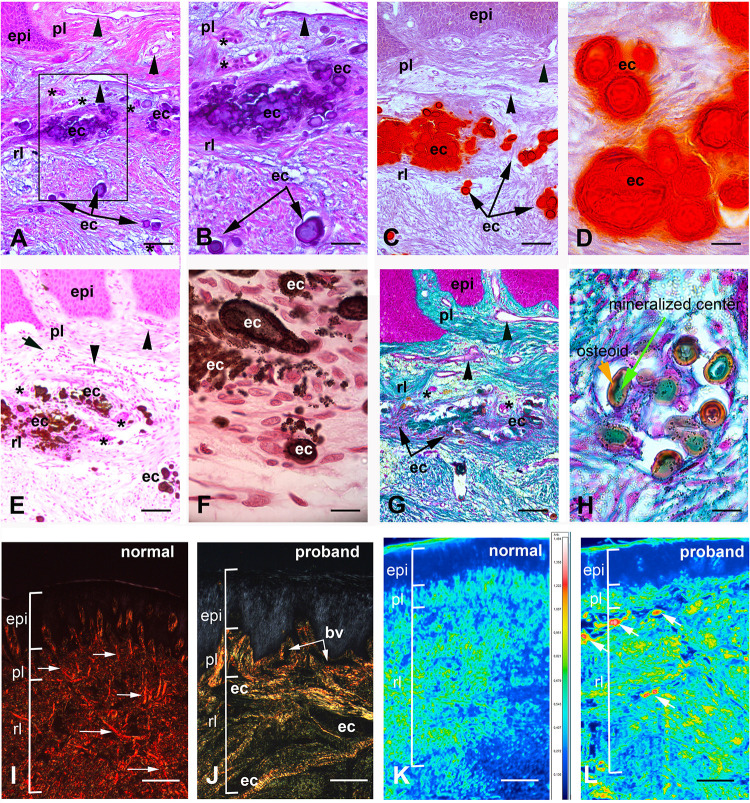
Histologic features of proband gingiva. **(A)** Hematoxylin-eosin staining shows numerous, tortuous capillary vessels with an abnormally large diameter in the papillary layer (arrowheads). Numerous ovoid calcified particles (ec) of various size are found in the reticular layer (rl) of the lamina propria. Epithelial islands (asterisks) are adjacent to the calcified particles. **(B)** Inset of **A** showing ovoid calcified particles in gingiva. **(C)** Alizarin red staining identifies calcium deposits in the reticular layer of the lamina propria. **(D)** High power photomicrograph showing that cells between calcified particles are heavily loaded in calcium. **(E,F)** Von Kossa staining indicates the presence of calcium phosphate. **(E)** Abnormal calcium deposits were located in the reticular layer of the lamina propria. Arrowheads indicate abnormally large blood vessels in the papillary layer (pl). **(F)** High power photomicrograph indicates the presence of cells intermixed with calcium deposits of various sizes. **(G,H)** Goldner Trichrome allows the detection of collagen in green. **(G)** In the papillary layer the light green coloration indicates a low amount of collagen. In the reticular layer collagen fibers do not assemble in large bundles and surround ectopic calcifications (ec). Arrowheads indicated abnormally large blood vessels and asterisks epithelial islands. **(H)** High power photomicrograph indicated that mineralized nodules (green) are surrounded by an osteoid-like layer (red/orange) **(I,J)** Picrosirius red polarizing staining. **(I)** Arrows indicate thick reddish bundles of collagen fibers in the normal gingiva lamina propria (rl and pl). **(J)** In the proband’s lamina propria, areas of loosely packed green–yellow disoriented collagen fibers and of yellow–red large collagen bundles were found. Asterisks indicate areas of no birefringence superimposing with calcified particles. Note the great diminution of collagen fibers in proband’s papillary layer. **(K,L)** F-TIR analysis at 1246 cm^–1^ indicates the presence of SO${}_{4}^{2-}$ residues in the extracellular matrix. In control gingiva **(K)** the IR spectra indicate a moderate to low concentration of SO${}_{4}^{2-}$ in the matrix. In proband gingiva **(L)** an increase of SO${}_{4}^{2-}$ residues is observed within the calcified areas (arrows). bv = blood vessel; epi = epithelium. Scale bars: **(A,C,E,G)** = 200 μm; **(B)** = 50 μm; (**D**,**H)** = 20 μm; **(F)** = 15 μm; (**I**,**J**) = 300 μm; (**K**,**L**) = 500 μm.

Histological analysis of the proband’s gingival tissue stained with hematoxylin-eosin showed a normally laminated epithelium. However, the epithelium was mildly acanthotic, the papillae appeared relatively large and their vascular network was overdeveloped (Figure 3A). The underlying connective tissue contained numerous randomly distributed ovoid calcifications, disorganized collagen bundles, clusters of presumably epithelial cells, fibroblasts and blood vessels (Figures 3A,B). Signs of inflammation were occasionally observed (Supplementary Figure 2A).

Laminated ovoid calcified structures of various sizes, positive for Alizarin Red and Von Kossa stains were observed throughout the connective tissue suggesting that the mineral deposits consisted of calcium phosphate complexes (Figures 3C–F). Interestingly, ovoid calcifications were intermixed with cells (Figures 3D,F). Goldner’s Masson trichrome further showed that the calcified particles were composed of a mineralized center (green) surrounded by an osteoid-like unmineralized extracellular matrix (ECM; red/orange) (Figures 3G,H).

To analyze collagen organization, we used picrosirius red staining of control and mutant gingiva. Picrosirius red polarizing staining showed a strong red birefringence in the normal lamina propria and closely packed thick collagen I fibers running principally from the cervical cementum to the interpapillary space (Figure 3I). Proband’s gingiva showed a different pattern with weakly birefringent, greenish fibers forming a loose network. Disorganized fibers were surrounding the epithelial islands and calcified structures. The greenish coloration also suggested a decrease in the ratio between collagen I and collagen III (Figure 3J and Supplementary Figure 2B,C).

Additional modifications in the gingival connective tissue were evidenced using Alcian blue staining of sulfated (pH 1) or carboxylated proteoglycans (pH 2.5) (Supplementary Figure 2D–I). An abnormally high abundance of both sulfated and carboxylated proteoglycans was observed in the proband’s sample. Fourier Transform infrared spectroscopic imaging analysis at 1246 cm**^–^**^1^ confirmed these observations, identified the presence of SO${}_{4}^{2}$**^–^** residues in the ECM and showed high amounts of sulfated proteoglycans at and near the calcifications of proband’s gingiva (Figures 3K,L).

### Collagen Modifications in the Mutant Gingiva

**FIGURE 4:**
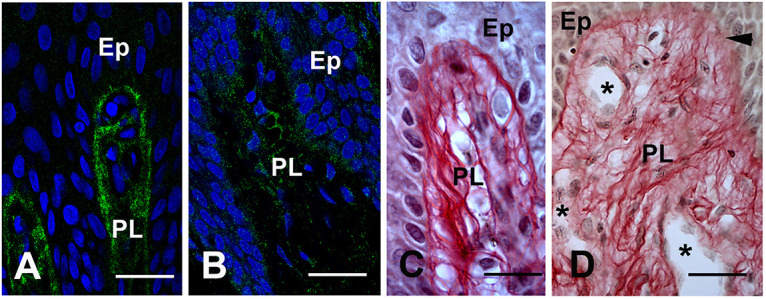
**(A)** COLLAGEN IV immunoreactivty in the basement membrane of the papillae (PL). **(B)** Punctuate patterns of COLLAGEN IV in proband gingiva. **(C)** Sirius red staining of the collagen network in the normal papillae. **(D)** Sirius Red staining in proband shows a disheveled network (arrowhead). Asterisks showed the tortuosity and abnormal diameters of vessels. Ep: Epithelium layer. Scale bars: **(A,B)** = 50 μm; **(C)** = 40 μm; **(D)** = 80 μm.

Under normal conditions the epithelial and connective tissue layers of the gingiva are separated by a COLLAGEN IV expressing basal lamina. The COLLAGEN IV signal was strong, homogenous and clearly demarcated the epithelial/connective tissue interface in control gingiva (Figure 4A). In the proband’s gingiva the signal of COLLAGEN IV was diffuse, had a punctuate pattern and indicated a disorganization of COLLAGEN IV in the basal lamina (Figure 4B). To detect collagen distribution in the papillary layer, we used Sirius red staining. In control gingiva high amounts of tightly packed, thick collagen fibers arranged in bundles were observed running the reticular layer of the lamina propria (Supplementary Figure 4A). In the papillary layers, thin collagen fibers, scattered or grouped in small bundles underlying gingival basement membrane or surrounding basement membranes of blood vessels (Figure 4C and Supplementary Figure 4A). Sirius red staining indicated a great disorganization of collagen distribution and assembly in proband’s gingiva. In the reticular layer, collagen fibers were not tightly packed in large bundles (Supplementary Figure 4B). Small bundles undulated beneath the papillary layer dispersing in contact with ectopic calcifications (Supplementary Figure 4B). In the papillary layer, collagen fibers did not assemble in small bundles but dispersed in a disheveled network (Figure 4D and Supplementary Figure 4B). These modifications may suggest that FAM20A activity is required for proper distribution and assemblage of collagen in the gingival connective tissue and the epithelial/connective tissue interface.

### Epithelial Islands in the Mutant Gingiva

FAM20A is strongly expressed in the gingival epithelium. Its function in this site is unclear, but *Keratin-14:Cre* driven epithelial ablation of FAM20A is sufficient to induce gingival overgrowth in mice (Li et al., 2016).

**FIGURE 5:**
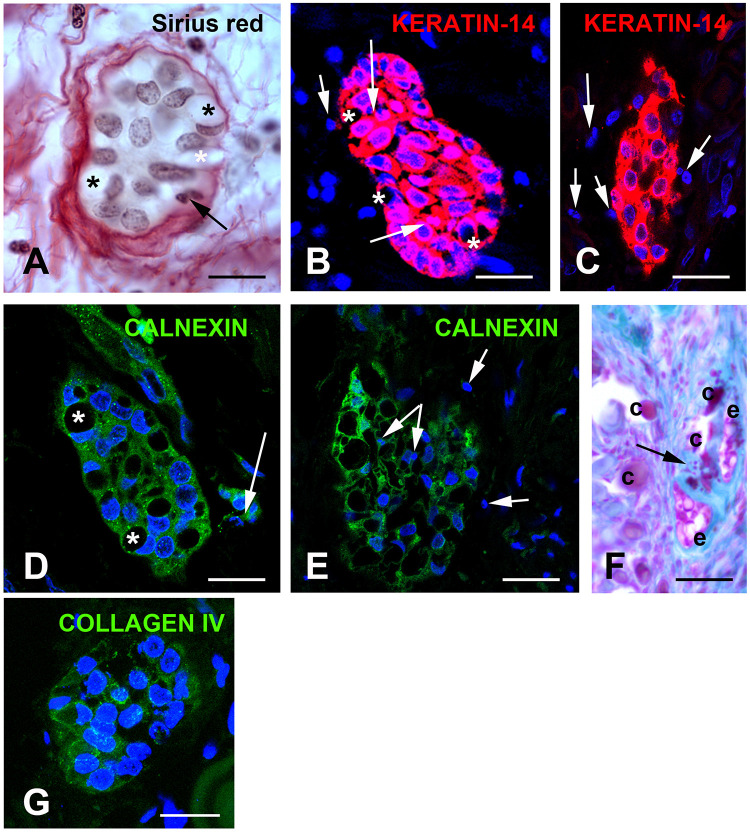
**(A)** Sirius red staining identifies clusters of cells with a yellowish cytoplasm typical of epithelial cell surrounded by loosely packed collagen fibers. Asterisks indicate large vacuoles associated with a crescent-shaped nucleus. Arrows indicate pyknotic nuclei. **(B,C)** KERATIN-14 (K14) immunostaining. **(B)** Little vacuolized islands strongly express K14. Arrows indicate pyknotic nuclei. **(C)** Increased vacuolization, loss of K14 and presence of numerous pyknotic nuclei (arrows). **(D,E)** CALNEXIN immunostaining suggests absence of endoplasmic reticulum in the vacuoles. **(D)** Vacuoles associated with crescent-shaped nuclei. **(E)** In highly vacuolized clusters, vacuoles fuse and numerous pyknotic nuclei are observed. Arrows indicate pyknotic nuclei. **(F)** Alcian Blue pH 2.5 staining indicates that epithelial and calcified particles are embedded in sulfated glycoproteins. Arrow indicated calcified nodules. **(G)** Absence of COLLAGEN IV immunoreactivity surrounding the epithelial islands. C, calcified nodules; e, epithelial island. Scale bars: **(A)** = 40 μm; (**B**,**D**,**G**) = 50 μm; **(C,E)** = 80 μm; **(F)** = 120 μm.

In proband gingiva, cell islands with epithelial morphology were observed in the subepithelial part close to the calcified nodules (Figures 3A–D). Cytosolic sirius red staining, a hallmark of epithelial cells (Figure 5A) and KERATIN-14 (K14) immunoreactivity confirmed the epithelial nature of these cells (Figures 5B,C). In some of these islands the cells displayed a crescent-shaped nucleus in contact with a large vacuole, in others numerous pyknotic nuclei were observed. It was interesting to note that a decrease in the K14 immunoreactivity was observed in cells with numerous vacuoles and pyknotic nuclei, suggesting an ongoing degenerative process (Figure 5C). This observation was further supported by the selective lack of CALNEXIN, a marker of the endoplasmic reticulum in the vacuolized areas (Figures 5D,E). Pyknotic bodies and various debris were found in a hyaline matrix composed of carboxylated and sulfated proteoglycans (Figure 5F). Finally, these islands were not surrounded by a COLLAGEN IV positive basal lamina, suggesting an odontogenic origin (Figure 5G).

### Connective Tissue Modifications in the Mutant Gingiva

#### Ultrastructural Analysis of the Gingival Nodules

**FIGURE 6:**
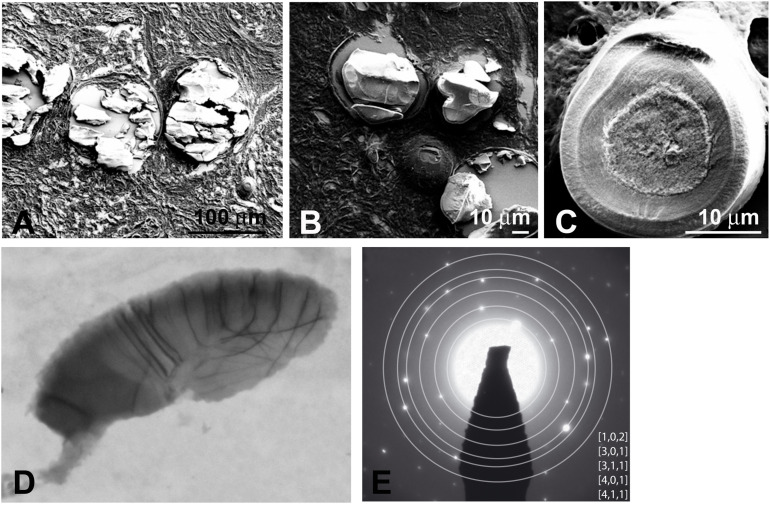
**(A,B)** SEM shows heavily mineralized entities in proband gingiva. **(C)** SEM of a mineralized sphericule displaying clear concentric layers in an inner coat around an amorphous nucleus. The outer coat is thick and displays a granular texture. TEM **(D)** and SAED pattern **(E)** of a small calcified sphericule. SAED indicates a hydroxyapathite-like material.

Mineralized nodules were observed throughout the lamina propria. Scanning electron microscopy (SEM) of proband’s gingiva identified heavily mineralized deposits of various sizes; 100 μm scale (Figure 6A), 30–60 μm scale (Figure 6B), and individual spherules/nodules larger than 10 μm (Figure 6C). It is possible that aggregates of the latter may form the mineralized deposits. The nodules formed concentric layers around a granular center (Figure 6C). Electron microscopy identified two major classes of nodules; class I from a 100 nm scale with weakly crystallized, stacked spherical structures (Supplementary Figure 4A) and class II (sphericule) with more mineralized larger nodules most likely resulting from a progressive fusion process (Figure 6D). Selected Area Electron Diffraction (SAED) of class I structures were devoid of rings’ pattern, suggesting a random crystallite arrangement (Supplementary Figure 4B). Selected Area Electron Diffraction showed that class II structures contained crystalline hydroxyapatite at the electron diffraction planes at (1 0 2), (3 0 1), (3 1 1), (401), and (4 1 1) (Figure 6E). The broken nature of the diffraction lines indicated the presence of a few crystallites (Figure 6E).

#### Expression of Pro-osteogenic and Vascular Factors in the ERS Gingiva

**FIGURE 7:**
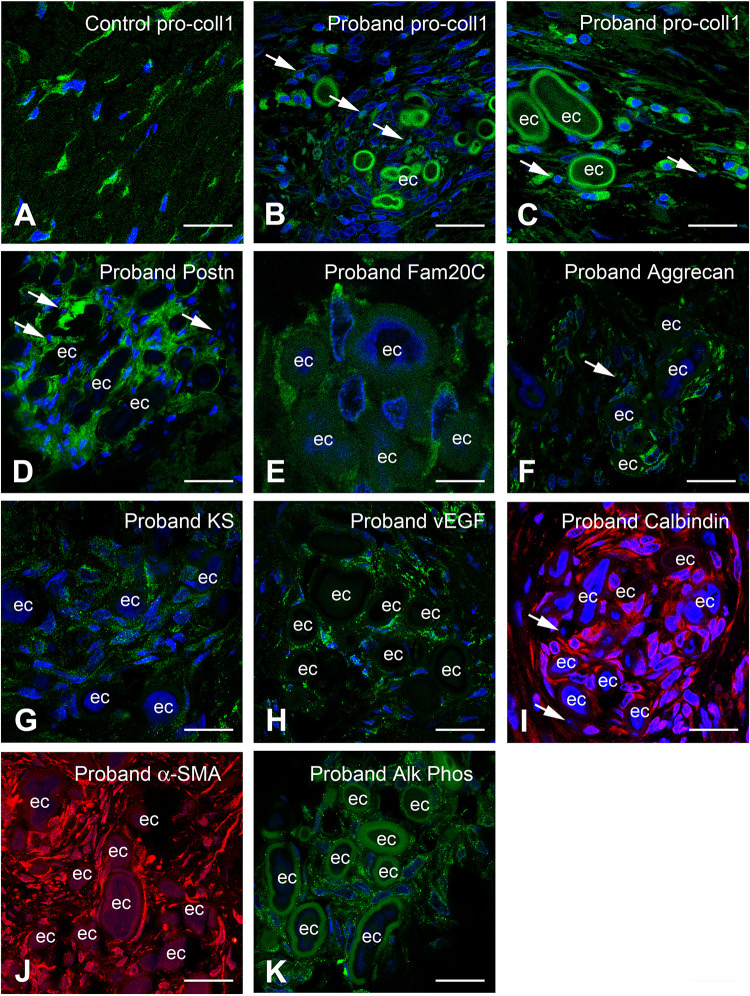
Immunocytochemical features of gingival calcifications. **(A)** Pro-COLLAGEN I expression in fibroblasts in normal gingiva. **(B,C)** In proband gingiva, pro-COLLAGEN I is expressed in cells with a round morphology, in the first external ring of calcified particles and small structures surrounding pyknotic bodies (thin arrows). **(D)** POSTN is localized in the extracellular matrix surrounding mineralized nodules and cells (thin arrows). **(E)** FAM20C is localized in the cytoplasm of cells associated with mineralized nodules. **(F)** ACAN, **(G)**, and Keratan sulfate expressions are associated with the extracellular matrix of gingival calcifications. Thin arrows in F indicated pyknotic body. **(H)** An abnormal expression of VEGF is seen within mineralized structures. **(I)** CALBINDIN and **(J)** α-SMA is strongly expressed in cells between the ectopic calcifications. Thin arrows in panel **(I)** indicated pyknotic bodies. **(K)** ALPL staining is also found as dots in cells between the ectopic calcifications. ec, ectopic calcification. Scale bars: (**A**–**D,F,H–K**) = 100 μm; **(E,G)** = 20 μm.

The calcified clusters were intermixed with or surrounded by cells having an ellipsoidal nucleus and acidophilic cytoplasm, suggesting that these cells were not fibroblasts. To characterize these cells and further investigate the gingival calcification process, we compared the expression of various chondrogenic/osteogenic-related markers in control and proband gingiva. In control gingiva COLLAGEN I propeptide antibody labeling cell-associated collagen I molecules, was detected solely in fibroblasts (Figure 7A). In proband’s gingiva pro-COLLAGEN I was found in fibroblasts, in round cells with short processes localized in the outer layer of the calcified particles and around cell debris (Figures 7B,C). In control gingival, PERIOSTIN (POSTN), a promoter of osteogenic differentiation was selectively found in the ECM of the papillary layer (Supplementary Figure 5A). In proband’s gingiva POSTN was strongly expressed in cells around the calcified nodules (Figure 7D) and so was FAM20C, a positive regulator of chondro/osteogenic maturation (Figure 7E). AGGRECAN (ACAN) is a large keratin sulfate (KS) substituted proteoglycan which has important properties in cartilage. ACAN and KS were not expressed in the connective tissue of the control gingival (Supplementary Figures 5B,C). In proband’s gingiva ACAN and KS were expressed in cells around calcified nodules (Figures 7F,G). Vascular Endothelium Growth Factor (VEGF), a critical regulator of angiogenesis and osteogenesis was only expressed in vascular cells in normal gingival (Supplementary Figure 5D) whereas in proband’s gingiva VEGF expression was found in cells intermixed with calcified nodules (Figure 7H). CALBINDIN, a calcification-associated anti-apoptotic factor expressed in gingival fibroblasts (Supplementary Figure 5E) was strongly detected in the cytoplasm of cells around calcified nodules (Figure 7I). Alpha-smooth myofibroblast actin (α-SMA) was normally expressed in vascular smooth cells and/or pericytes of lamina propria gingival blood vessels (Supplementary Figure 5F). In the proband’s gingiva α-SMA was also readily detected in cells of the calcified areas (Figure 7J). Finally, the tissue-nonspecific ALKALINE PHOSPHATASE (ALPL) was detected in cells around and intermixed with calcified nodules (Figure 7K and Supplementary Figure 5G). All together the above observations strongly suggest that the lack of FAM20A is associated with *de novo* expression of chondro/osteogenic characters by cells surrounding and intermixed with calcified nodules.

### Osteogenic Trans-Differentiation of FAM20A Deficient Gingival Fibroblasts

To investigate the potential implication of FAM20A in the induction of osteogenic characters we isolated control and mutant gingival fibroblasts. After collagenase digestion and dissociation of the epithelial layer, primary control and the proband’s gingival fibroblasts (GFs) were cultured at passage 2 for a total period of 21 days in standard or mineralization-inducing medium.

**FIGURE 8:**
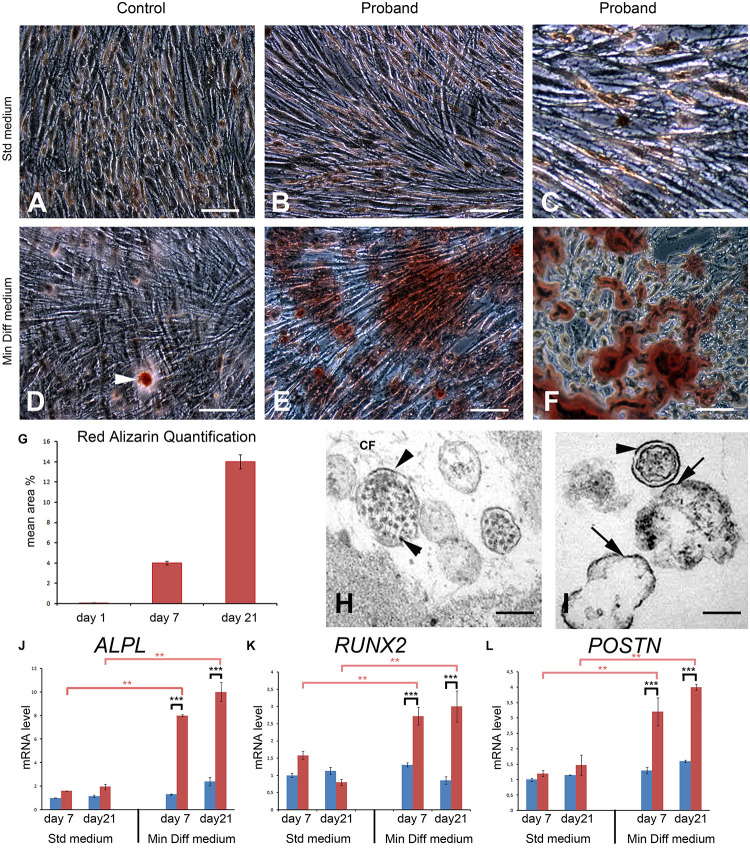
An osteogenic-like process is activated in FAM20A deficient gingival fibroblasts (GFs) grown in mineralization-inducing conditions. **(A,B)** Representative bright field images of normal **(A)** and FAM20A deficient **(B,C)** GFs grown for 21 days in standard medium colored with Red Alizarin. **(D–F)** Representative bright field images of normal **(D)** and FAM20A deficient **(E,F)** GFs grown 21 days in a mineralization-inducing medium colored with Red Alizarin. **(D)** Arrowhead indicates rare and small calcium deposits in control GFs. **(E)** Calcium deposits of various sizes are visualized in FAM20A deficient cultures. **(F)** High power photomicrograph of calcium deposits intermixed with cells. **(G)** Quantitative analysis of Red Alizarin expression by morphometric analysis on the pooled results of three separate experiments; the value represents the means ± SD. *p < 0.05. **(H,I)** Transmission electron microscopy images showing extracellular matrix vesicles containing electron dense bodies. Initial calcium-phosphate deposition takes place at the interior membrane (arrows). Depositions are also observed around cell debris. **(J–L)** qRT/PCR of *ALPL* **(J)**, *RUNX2* **(K)**, and *POSTN* **(L)** at 7 and 21 days of culture. Data are presented as the mean ± SD of three separate experiments. ***p < 0.001. Scale bars: (**A**–**F**) = 150 μm; (**H**,**I**) = 2 μm.

In standard conditions the control and proband’s GFs formed a homogeneous monolayer. At day 21, both cultures became highly confluent and GFs displayed long spindle shaped morphologies typical of fibroblast-like cells. Alizarin red staining did not reveal any deposits in either culture (Figures 8A–C). Control GFs grown in the mineralization-inducing medium did not modify their morphology. Occasionally small calcium deposits could be seen in these cells after 21 days in culture (Figure 8D). Under the same conditions the FAM20A deficient GFs formed dense deposits of calcium aggregates (Figures 8E,F). Morphometric analysis showed a time dependence trend, with significantly increasing calcium deposition between 7 and 21 days of culture (Figure 8G). Ultrastructural analysis revealed the presence of extracellular vesicles with a diameter of 100–300 nm near the mutant cells (Figures 8H,I). Electron dense bodies and membrane deposition were observed resembling to mineral nucleation (Figure 8H). Mineral nucleation was also observed around cell debris (Figure 8I). The expression of osteogenic genes like *RUNX2*, *ALPL* (early osteogenic programming genes), *POSTN* (later osteogenic programming gene), as well as the α1 and α2 chain of COLLAGEN type I (*COL1A1* and *COL1A2*) was evaluated using qRT/PCR at 7 and 21 days of culture. No statistical differences were observed in standard conditions. In mineralization-inducing conditions statistical differences emerged in *ALPL*, *RUNX2* and *POSTN* expression as soon as day seven. The upregulation of *ALPL*, *RUNX2* and *POSTN* mRNA was further amplified until day 21 (Figures 8J–L). The *COL1A1*-to-*COL1A2* mRNA ratio can be used as an indicator of mineralization (Couchourel et al., 2009). Our results showed that proband’s gingival fibroblasts produced a matrix with a decreased mRNA ratio of *COL1A1* to *COL1A2* (0.8-fold lower by day 7 and 0.45-fold lower by day 21; Supplementary Figure 6), a condition prone to mineralization. These results were also consistent with the immunohistochemical data. They clearly suggest that the lack of FAM20A may trigger osteogenic-like cell modifications by day seven and onward.

## Discussion

In this study we reported an ERS patient with extreme oligodontia, tooth agenesis, hypoplastic amelogenesis imperfecta, severe gingival overgrowth and ectopic calcifications including in the gingiva, the dental pulp, the kidney and the proximity of the cervical and thoracic vertebrae. We identified a new homozygous point mutation in exon 1 resulting in a premature stop codon and complete lack of FAM20A protein. We focused on the gingival phenotype and studied the cellular and ultrastructural modifications associated with the absence of FAM20A. The proband’s gingiva was highly vascularized and contained numerous clusters of epithelial cells as well as calcified nodules. In line with the morphological and biochemical modifications, the expression of chondro/osteogenic markers was increased near and within the nodules. *In vitro*, FAM20A-deficient fibroblasts were prone to calcify under mineralization-inducing conditions. This was associated with the formation of extracellular vesicles and the upregulation of known osteogenic markers including *ALP*L, *RUNX2* or *POSTN*. Our study highlights some aspects of ERS pathogenesis and suggests that FAM20A acts locally to prevent ectopic calcification in the gingiva.

Gingival overgrowth of variable severity is a typical, macroscopic characteristic of ERS gingiva. Microscopic findings described in previous studies include increased amounts of randomly arranged collagen bundles and calcified nodules in close proximity to odontogenic epithelium cells in the connective tissue (O’Sullivan et al., 2011; Cho et al., 2012; Jaureguiberry et al., 2012; de la Dure-Molla et al., 2014; Kantaputra et al., 2017; Pêgo et al., 2017; Wang et al., 2019). The clinical and histological findings reported here were consistent, albeit more pronounced, with these observations. No clear genotype-phenotype correlations are currently available for ERS patients; it is possible, however, that the lack of FAM20A may explain the severity of the oral phenotype presented here.

FAM20A is a secreted protein that in addition to teeth and kidney is expressed in hematopoietic cells, lung, liver, and chondrocytes (Nalbant et al., 2005; Azuma et al., 2015). In the latter FAM20A was proposed to affect ECM biosynthesis, including of collagen, an effect that may be independent on FAM20C or FAM20B (Azuma et al., 2015). Recent data also indicate that FAM20A would not be an active kinase but an allosteric activator of FAM20C (Cui et al., 2015). Furthermore, FAM20A favors the extracellular secretion of FAM20C in mouse embryonic fibroblasts (Ohyama et al., 2016). It has also been proposed that extracellular FAM20C sustains mineralization sustains mineralization of osteoblast cells (MC3T3) *in vitro* (Ohyama et al., 2016). This is partly in agreement with the bone osteosclerosis displayed by patients suffering RNS, which is a syndrome caused by the lack or reduced Fam20C activity (Sheth et al., 2018; Mameli et al., 2020). Indeed, bone osteosclerosis could be related to the lack or reduced activity of extracellular FAM20C in the developing osteoid. Surprisingly, ERS patients do not suffer from constitutional bone diseases (Jaureguiberry et al., 2012) such as osteosclerosis indicating that FAM20A does not participate predominantly in the secretion of FAM20C in the developing bone. Importantly, gingival and pulpal ectopic calcifications have been reported in non letal RNS patients (Acevedo et al., 2015). Taken together, this suggests that the lack of FAM20A or the reduced/lack FAM20C activity in the gingiva supports the formation of ectopic calcified nodules. This seems in contradiction with the idea of a mineralization occurring with extracellular FAM20C. However, careful considerations have to be taken since mechanisms that lead to pathological ectopic calcifications, are probably very different from those involved in physiological mineralization. The nature of the FAM20A/FAM20C interactions in fibroblasts and the consequence of the reduced/lack of FAM20C activity on gingival fibroblast behavior (trans-differentiation) under mineralization conditions are open questions that need to be solve.

How FAM20A triggers modifications of ECM biosynthesis/composition is an open question. We showed that in addition to collagen disorganization, the proteoglycan composition of the FAM20A deficient gingiva was dramatically altered. The expression of carboxylated and sulfated proteoglycans, typically ascertained by Alcian blue staining and FTIR analysis, was much more pronounced in the proband compared to controls. This change was accentuated around mineralized nodules. ACAN, the major proteoglycan of the articular cartilage (Kiani et al., 2002), was strongly expressed in the nodules of proband but never observed in control gingiva. ACAN carries numerous KS and chondroitin sulfate chains, an enormous amount of negative charges and regulates cell adhesion. Overexpression of aggrecan may thus be disruptive to ECM integrity and potentially promote retention of mineral. Supporting this hypothesis, ACAN upregulation has been associated with vascular calcification and chronic kidney disease (Neven et al., 2010; Chen and Simmons, 2011).

We also showed that in the ERS gingival lamina propria, epithelial cell islands in the vicinity of growing mineralized nodules progressively degenerated. These cells displayed crescent-shape nuclei with one huge vacuole in the cytoplasm. As the degeneration progressed cells lost K14 expression, nuclei fragmented and cellular debris were found at the periphery. Interestingly, epithelial islands were not surrounded by the principal basal lamina protein COLLAGEN IV.

Our hypothesis is that the absence or dysfunction of FAM20A causes/contributes to the degeneration of gingival epithelial cells. Calcified deposits appeared near degenerating cells, suggesting that early calcification was linked to epithelial cell death, and pointing to these areas as nidus for the process. Near and within calcifications fibroblasts were undetectable but cells with a chondrogenic/osteogenic phenotype were readily found: they expressed a panel of specific markers including α-SMA, a marker of osteoprogenitor cells that have the potential to differentiate into mature osteoblasts (Kalajzic et al., 2008). It is therefore likely that, like in vascular calcification, phosphate and calcium precipitation in the gingiva is the outcome of a complex series of events involving both cell death and trans-differentiation.

Proband’s GFs readily transdifferentiated into an osteogenic phenotype in mineralization-inducing conditions. It is of interest that this process was accompanied by the formation of extracellular, presumably matrix vesicles known to initiate normal and pathological mineralization (Hasegawa et al., 2017). *ALPL*, *RUNX2*, and *POSTN*, genes involved in osteoblast differentiation were upregulated in the mineralization-inducing medium already at day 7, most likely prior to the formation of calcified deposits or the identification of pyknotic profiles, and continued to increase until day 21. These observations might suggest that cell death is not a prerequisite for ectopic mineralization and point to a local effect of FAM20A in the calcification process.

Mineral metabolism is regulated by various factors including vitamin D3 and PTH. In addition, a balance of serum calcium and phosphate is necessary to avoid the formation of calcium phosphate crystals. Whereas the PTH levels were normal, the blood phosphorus was increased in the proband suggesting high intake or renal dysfunction. Additionally, both kidneys showed signs of calcification despite a still normal glomerular filtration rate. It is unclear how FAM20A may cause hyperphosphatemia, a major risk factor for chronic kidney disease, but our data are compatible with ERS-associated renal dysfunction or failure.

We cannot exclude that a local dysregulation of calcium homeostasis leading to oversaturation of calcium phosphate salts and spontaneous hydroxyapatite crystallization may contribute to the gingival calcification. Nevertheless, the results presented here strongly support the idea that, like in other soft tissues, gingival calcification caused by FAM20A dysfunction is an organized biomineralization process involving chondro/osteogenic trans-differentiation of fibroblastic cells. We therefore assume that FAM20A functions, among others, to prevent ectopic calcification in the gingiva and most likely other FAM20A expressing tissues.

## Data Availability Statement

The original contributions presented in the study are included in the article/Supplementary Materials, further inquiries can be directed to the corresponding author.

## Ethics Statement

The studies involving human participants were reviewed and approved by CODECOH DC-2018-3382 INSERM. Written informed consent to participate in this study was provided by the participants’ legal guardian/next of kin.

## Author Contributions

AB and RK conceived the study. CC, MV, OC, and MD-M participated in the design of the study. VSE, OC, AB, and RK wrote the manuscript. VSE, AB, and AN performed the experiments. AD, RF, and MQ analyzed the data. VC participated in histological study. OC, AB, MD-M, and RK contributed equally to the work. All authors contributed to the article and approved the submitted version.

## Conflict of Interest

The authors declare that the research was conducted in the absence of any commercial or financial relationships that could be construed as a potential conflict of interest.
