# Supplementary material for: Lack of FAM20A, Ectopic Gingival Mineralization and Chondro/Osteogenic Modifications in Enamel Renal Syndrome
Source: Front Cell Dev Biol. 2020 Dec 8;8:605084. doi: 10.3389/fcell.2020.605084 (PMC7793853; doi:10.3389/fcell.2020.605084)
Supplement: Supplementary file 1 [file Data_Sheet_1.PDF]

Supplemental Informations to

Lack of FAM20A, ectopic gingival mineralization and chondro/osteogenic modifications in Enamel Renal syndrome

Victor Hugo Simancas[1], Adrien Naveau[1], Abdoulaziz Diarra[1], Arnaud Dessombz[1], Rufino Felizardo[2], Christos Chatziantoniou[3], Mickaël Quentric[4], Miika Vikkula[4], Olivier Cases[1], Ariane Berdal[1,2], Muriel De La Dure-Molla[2,5], Renata Kozyraki[1,2].

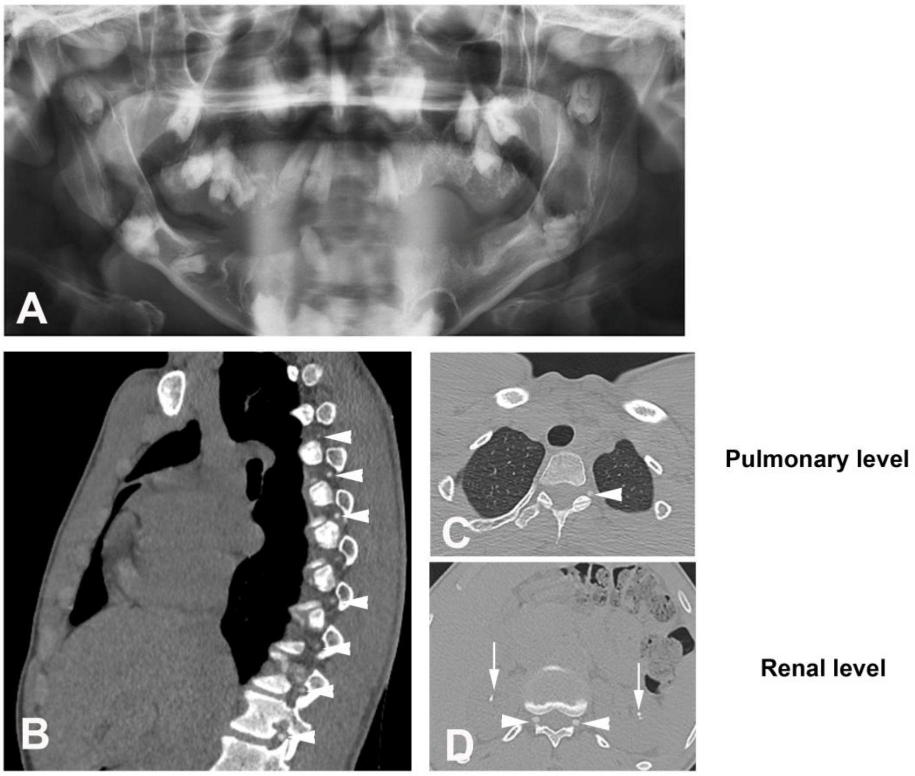

Supplemental Figure 1: **(A)** Panoramic radiograph of proband. (**B-D**) CT scans of proband. **(B**) Sagittal section showing abnormal circular calcifications along the spine in all vertebral foramens (arrowheads). (**C, D**) Coronal sections at the level of lungs (**C**) and kidneys (**D**). Arrowheads indicated abnormal calcifications and arrows nephrocalcinosis.

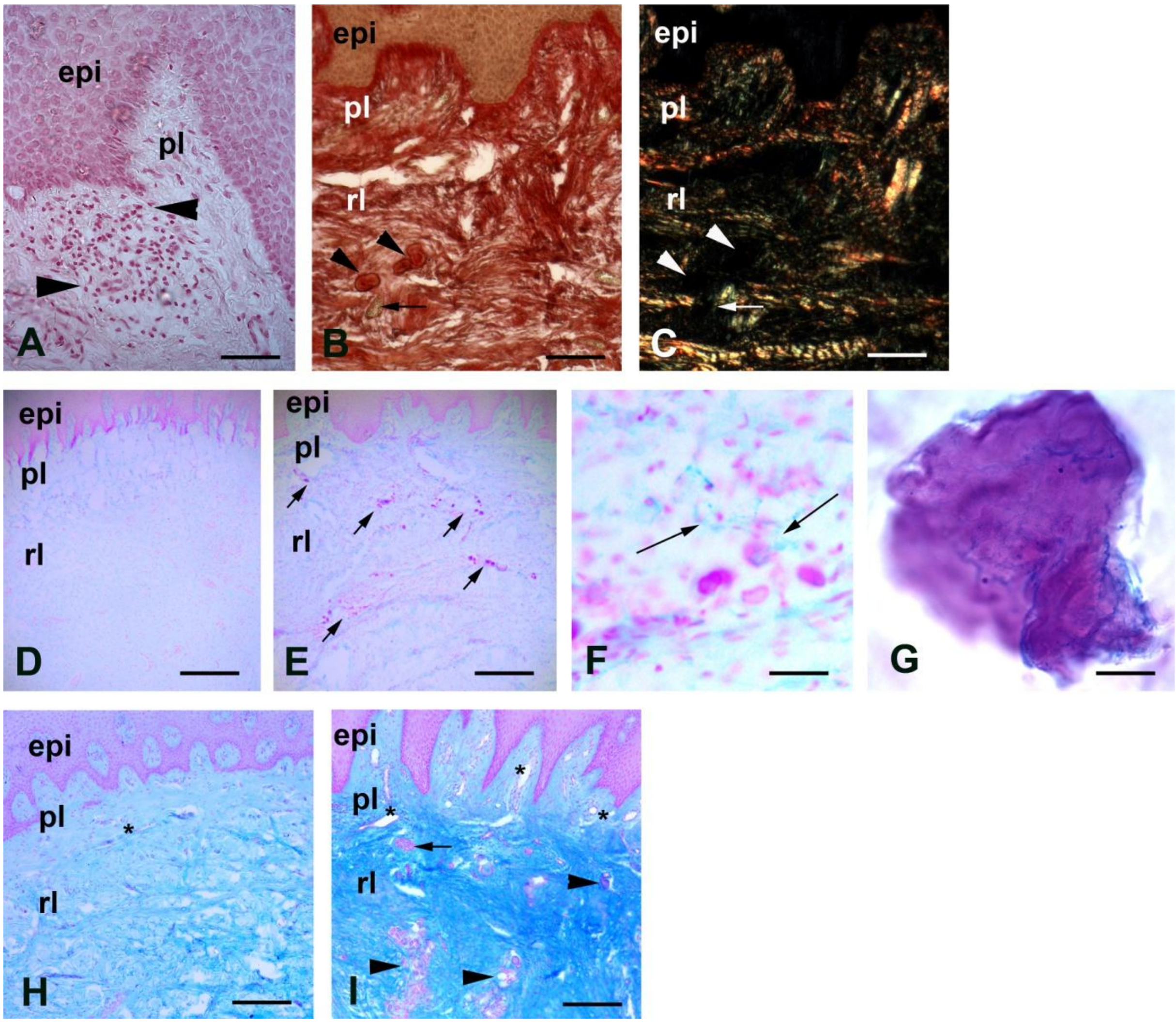

Supplemental Figure 2: (**A**) Red Nucleus staining identified large clusters (between arrowheads) of mononuclear inflammatory cells in the papillary layer (pl) next to the epithelial layer (epi). **(B)** Picrosirius red staining in proband. Arrowheads indicated ectopic calcifications. Thin arrow indicated an island of epithelial cells. **(C)** Polarized picrosirius red staining of the same section. The predominance of greenish coloration indicated a diminution in collagen I contribution. Arrowheads indicated ectopic calcifications. Thin arrow indicated an island of epithelial cells. (**D-G**) The alcian blue stain (pH 1.0) visualized strongly sulfated polysaccharide chains of core proteins. (**D**) In control section, staining was weak in the papillary layer and the reticular layer (rl) of the lamina propria. (**E**) In proband gingiva, an increased staining was found near ectopic calcifications (arrows). (**F**) A network of strongly stained filaments (arrows) was found in the vicinity of calcifications. (**G**) A web of blue staining wrapped ectopic calcification. (**H-I**) The alcian blue stain (pH 2.5) visualized

carboxylated polysaccharide chains of core proteins. (**H**) In control gingiva, the staining was uniform in the lamina propria. (**I**) The staining was particularly increased in the reticular layer of the proband. Asterisks indicated blood vessels. Arrowheads indicated ectopic calcifications. Thin arrow indicated an island of epithelial cells. Scale bars: A = 120 μm; B, C = 200 μm; D, E, H, I = 400 μm; F = 80 μm; G = 20 μm.

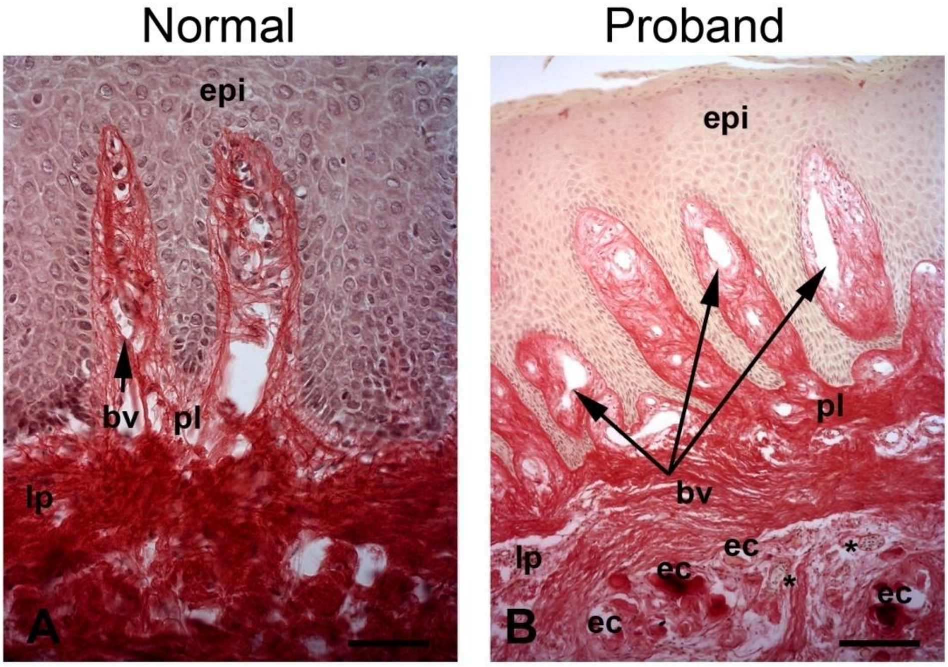

Supplemental Figure 3: Picrosirius red stains collagen. (**A**) In the normal upper part of the reticular layer of the lamina propria (lp) the connective tissue was composed of large, dense bundles of long thick collagen fibers running tangentially to the epithelial layer. The connective tissue of the papillary layer was composed of short, thin collagen fibers, scattered or grouped in small bundles running near the epithelio-connective basement membrane and blood vessel (bv). (**B**) In the reticular layer of proband, the connective tissue was greatly disorganized: collagen fibers did not firmly held to form dense bundles, undulated between ectopic calcifications (ec) and swirled around epithelial islands (asterisks). In the papillary layer, the connective tissue was composed of loose scattered collagen fibers forming a fine meshwork. bv: blood vessel. Scale bars: 150 μm.

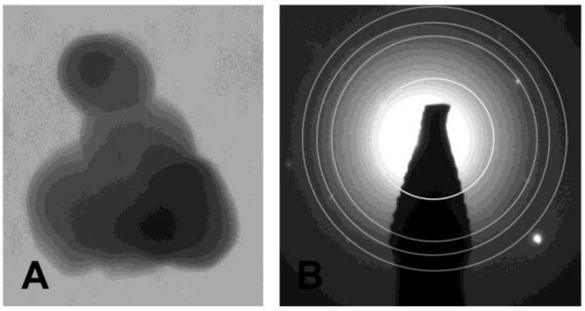

Supplemental Figure 4: TEM (**A**) and SAED pattern (**B**) of a very small calcified particle without rings structure.

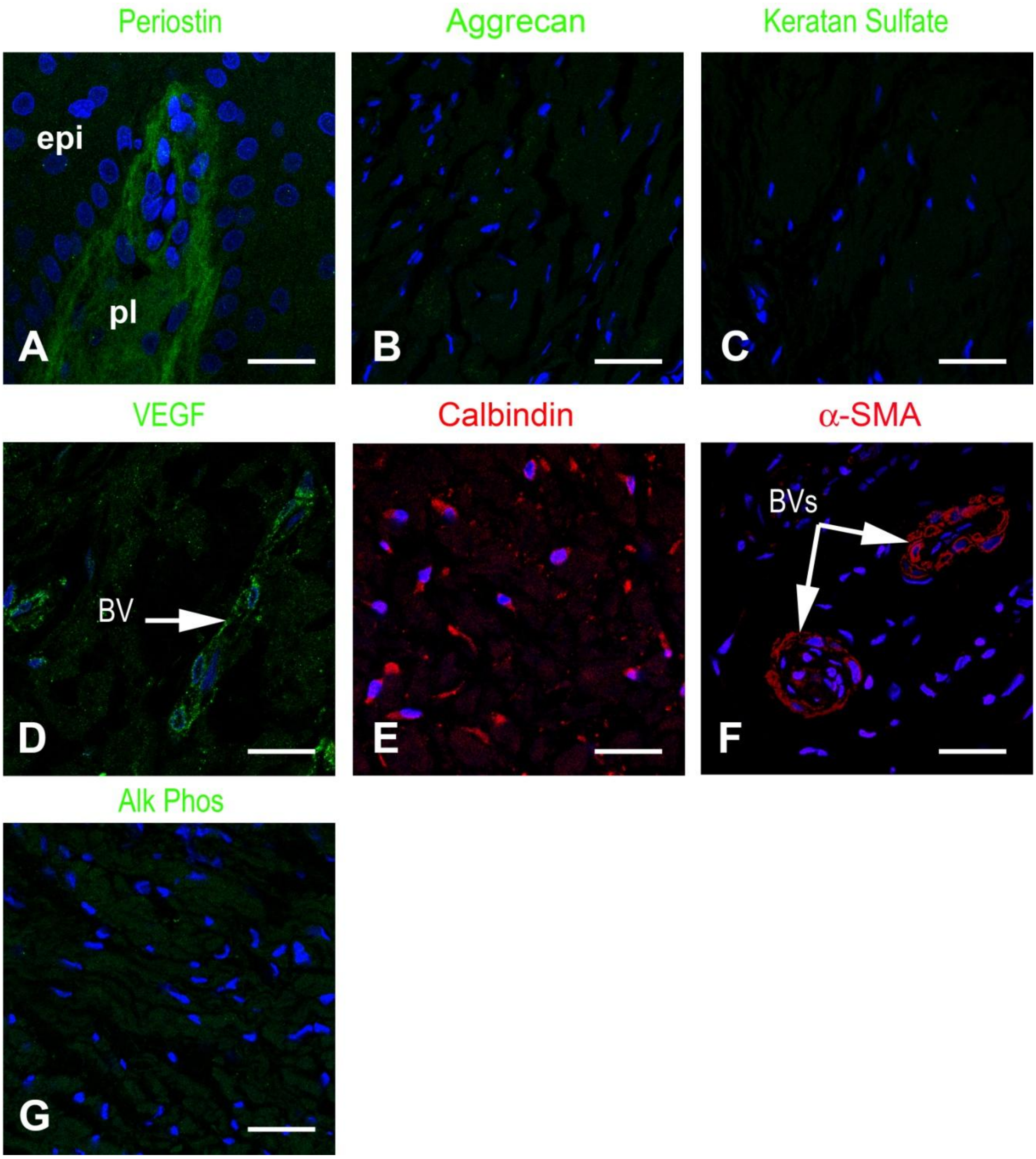

Supplemental Figure 5: Expression of selective markers in the control gingiva. (**A**) POSTN immunoreactivity (IR) was selectively found in the extracellular matrix of the papillary layer (pl). (**B**, **C**) ACAN (**B**) and KS (**C**) IRs were not detected in the gingival connective tissue. (**D**) Vascular endothelial growth factor (VEGF) IR was localized in gingival blood vessels (BVs). (**E**) CALBINDIN expression was found in fibroblasts. (**F**) α-SMA was expressed by pericytes of gingival blood vessels. (**G**) Alkaline phosphatase was not detected in the gingival connective tissue. epi: gingival epithelium. Scale bars: A= μm; B, C, E, F, G= μm; D= μm.

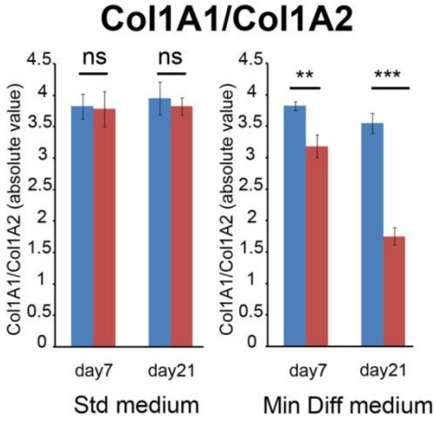

Supplemental Figure 6: Ratios of mRNA expression levels of *COLLAGEN type 1 A1* (*COL1A1*) to *COLLAGEN type 1 A2* (*COL1A2*) between normal (blue bars) and proband (red bars) gingival fibroblastic cultures under standard or mineralization conditions at day 7 and day 21. ns = not significant; **=p<0.05; ***=p=<0.001.
